# History, origin, and function of transzonal projections: the bridges of communication between the oocyte and its environment

**DOI:** 10.21451/1984-3143-AR2018-0061

**Published:** 2018-08-16

**Authors:** Hugh J. Clarke

**Affiliations:** 1 Department of Obstetrics and Gynecology, McGill University, Montréal, QC, Canada.; 2 Research Institute, McGill University Health Centre, Montreal, QC, Canada.

**Keywords:** communication, filopodia, follicle, granulosa cell, oocyte

## Abstract

Development and differentiation of a functional oocyte that following fertilization is able to give rise to a new individual requires continuous physical contact with the supporting somatic cells of the ovarian follicle. As the oocyte is surrounded by a thick extracellular coat, termed the *zona pellucida*, this essential contact is mediated through thin cytoplasmic filaments known as transzonal projections (TZPs) that project from the somatic granulosa cells adjacent to the oocyte and penetrate through the *zona pellucida* to reach the oocyte. Gap junctions assembled where the tips of the TZPs contact the oocyte plasma membrane, and other contact-dependent signaling may also occur at these sites. Here, I describe early studies of TZPs, which were first identified in the late 19th century, discuss their similarities with classical filopodia, review their structure and function, and compare two models that could account for their origin. Possible priorities and directions for future studies close this contribution.

## Introduction

**Figure 1:**
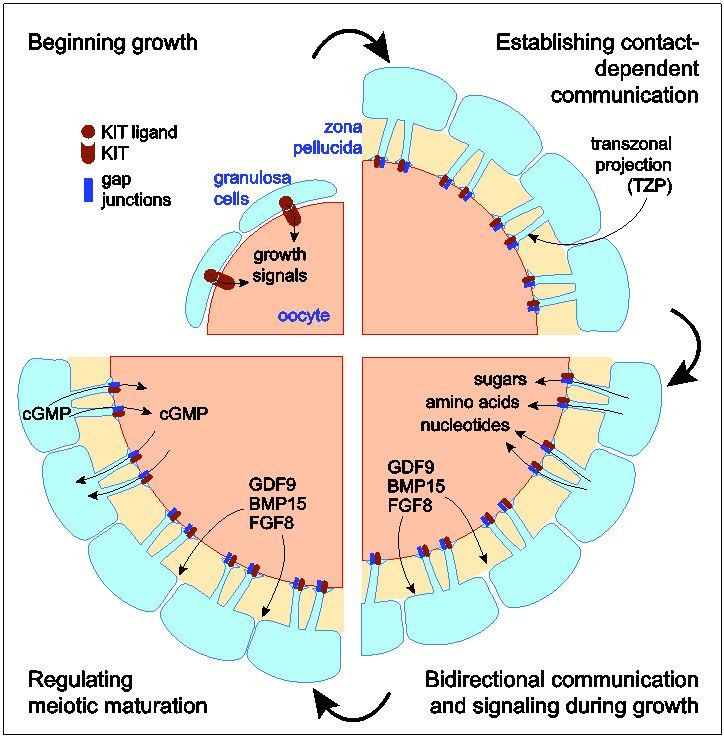
Communication with the somatic environment regulates all aspects of oocyte development. Clockwise from upper left: KIT ligand produced by the granulosa cells, which activates the KIT receptor on the oocyte plasma membrane, is thought to initiate growth of oocytes within primordial follicles. As the growing oocyte elaborates the *zona pellucida *, separating it from the bodies of the granulosa cells, the two cell types retain contact via transzonal projections (TZPs) that traverse the extracellular coat. Gap junctions, and potentially membrane-associated KIT ligand, are present where the tips of the TZPs contact the oocyte plasma membrane. The gap junctions permit the granulosa cells to transfer small molecules (<1 kDa) to the growing oocyte. In a complementary manner, the oocyte secretes soluble growth factors that promote metabolic activity and differentiation of the granulosa cells. As oocytes reach full-size, cGMP produced by the granulosa cells is transferred to the oocyte where it prevents meiotic maturation. Conversely, maturation is initiated when cGMP levels in the granulosa cells fall so that cGMP returns to them from the oocyte, thus causing the cGMP level in the oocyte to fall. See text for further details.

Germ cell differentiation depends on communication with the somatic cells of the gonad, which constitute a micro-environment that provides it with essential nutrients and regulatory signals at each stage of its development ( Fig. 1 ; El-Hayek and Clarke, 2016 ; Clarke, 2017a ). In mammalian females, the granulosa cells of primordial follicles are thought to send signals that trigger the oocyte within to begin to grow. In growing follicles, the granulosa cells provide the oocyte with essential metabolites and send a signal that prevents the precocious initiation of meiotic maturation. Finally, during maturation, the granulosa cells provide still unidentified factors that increase protein synthesis in the germ cell. This intercellular communication must be established and maintained in the face of a daunting barrier - a thick extracellular coat, termed the *zona pellucida*, that surrounds the oocyte and physically separates it from the bodies of the granulosa cells. To overcome this barrier, thin cytoplasmic processes extend from the granulosa cells through the *zona pellucida* to the oocyte surface. These transzonal projections (TZPs) constitute the only means of contact and contact-dependent communication between the oocyte and its somatic environment and thus are indispensable for the production of an oocyte that is able to give rise to a new individual. Here, I review early studies of TZPs and cognate structures in non-mammalian species, describe their nature and function, discuss different models that may explain their origin, and conclude with questions for future study.

## Discovery and description of TZPs

**Figure 2:**
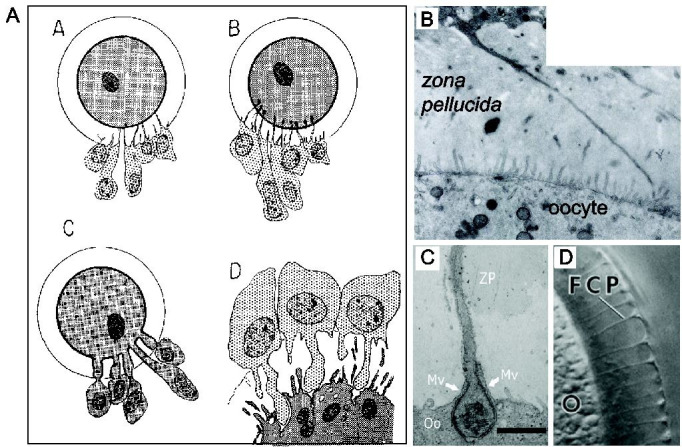
Identification of TZPs. A) Early depictions of TZPs and their relationship with the oocyte as described in ( Hadek, 1965 ). Please see this review for citations of the original manuscripts. Nagel (1888, panel A) suggested that contents of the granulosa cells travel through channels within the *zona pellucida* and are then poured into the perivitelline space. Mjassojedoff (1923, panel B) proposed that the TZPs terminate within the oocyte. Duryee (1954, panel C) concluded that there is likely cytoplasmic continuity between the oocyte and granulosa cells. Panel D shows the author’s interpretation, which essentially matches our current understanding. Reproduced with permission. B) Electron micrograph traces a TZP from its origin at the granulosa cell to the surface of the oocyte. Many microvilli can be seen at the oocyte surface. Reproduced with permission from ( Zamboni, 1974 ). C) TZPs tips frequently become embedded in pockets at the oocyte surface and partially enveloped by protrusions of the oocyte membrane. Reproduced with permission from ( Macaulay *et al*., 2014 ). D) Follicle cell processes (FCP) traverse the vitelline membrane of non-mammalian oocytes – in this vase the starfish – to reach the cell surface. Reproduced with permission from ( Schroeder, 1981 ).

The first recorded descriptions of TZPs date from the late 19th century ( Fig. 2A ), where they were identified using light microscopy as birefringent channels in the *zona pellucida* ( Hadek, 1965 ). Their probable role in nourishing the growing oocyte was immediately recognized and several potential mechanisms were proposed, including that they poured their contents into channels in the *zona pellucida* that presumably conducted them to the oocyte; that they established a cytoplasmic continuum with the egg thereby producing a giant multinucleate cell; and that they made contact with oocyte surface but the plasma membranes of the two cell types remained intact ( Hadek, 1965 ). Thus, even at this relatively early stage, scarcely 50 years after the mammalian oocyte had first been observed, the mechanism and importance of interacting with its environment had been inferred.

Rapid progress in identifying and characterizing TZPs came with the development of electron microscopy. A large number of papers, notably including though not restricted to the laboratories of Everett Anderson, Keith Porter and Roberto Sotelo, established that TZPs are filamentous projections that extend from the granulosa cells immediately adjacent to the oocyte and penetrate through the entire width of the *zona pellucida* to reach the oocyte surface ( Fig. 2B ; Trujillo-Cenoz and Sotelo, 1959 ; Anderson and Beams, 1960 ; Anderson *et al*., 1976 ). There, the tip of the TZP often spreads to form a bulbous or foot-like structure that were partially enveloped (or hugged) by protrusions of the oocyte ( Fig. 2C ; Macaulay *et al*., 2014 ), thereby increasing the area of surface contact between the two cells. Both gap junctions, discussed in more detail below, and tight junctions are present at the sites of plasma membrane apposition ( Anderson and Albertini, 1976 ; Gilula *et al*., 1978 ). Multiple TZPs project from a single granulosa cell – as many as 70 have been reported ( Makabe *et al*., 2006 ) – apparently originating from one or a small number of central hubs or foci. TZPs have been described in a wide range of mammals ( De Smedt and Szollosi, 1991 ; De Lesegno *et al*., 2008 ). In primates, TZPs have been identified that project deep into the oocyte, reaching even as far as the nucleus ( Hope, 1965 ; Motta *et al*., 1994 ; Makabe *et al*., 2006 ). Studies such as these unequivocally established the nature of the germ line-soma interaction.

Inside the TZPs, the early studies revealed long filamentous striations running parallel to its axis. In addition, organelles such as mitochondria were also found along the shaft or at the base of TZPs, although what fraction of TZPs that contained such structures was generally not reported and would have been difficult to determine using electron microscopy. Between the tip of the TZP and the oocyte plasma membrane, extracellular vesicles can sometimes also be seen ( Macaulay *et al*., 2014 ). Although the fraction of TZPs that are associated with vesicles is not known, this identifies a potential mechanism by which large cargo including organelles might be transferred from the granulosa cells to the oocyte.

Oocytes of non-mammalian species are also enclosed by an extracellular coat, often termed a vitelline membrane ( Fig. 2D ). Electron microscopy has revealed microfilament-rich processes emanating from the follicle cells that traverse the vitelline membrane to reach the oocyte surface in organisms including frog, newt, chicken and starfish ( Hope *et al*., 1963 ; Perry *et al*., 1978 ; Schroeder, 1981 ; Browne and Werner, 1984 ). As in mammals, they frequently terminate as bulbous swellings that occupy pouches in the oocyte surface and gap junctions can be seen where the two membranes are closely apposed. An intriguing exception to this pattern is the zebrafish, in which both the follicle cell processes and long microvilli extending from the oocyte pass through pores in the vitelline envelope to enable direct cell-cell contact. Moreover, the oocyte microvilli appear to play a more important role in mediating the contact ( Kessel *et al*., 1985 ). These observations show that the principle of germ line-somatic contact through filamentous projections is highly conserved evolutionarily.

## Structure of TZPs

**Figure 3:**
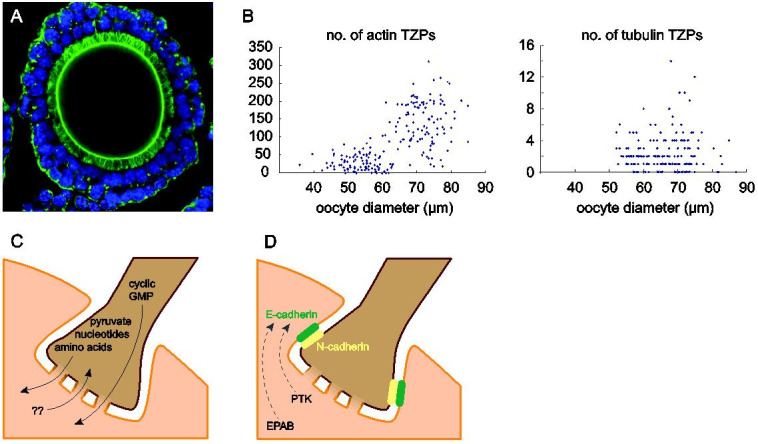
Characterization of TZPs. A) Confocal optical section of a granulosa cell-oocyte complex of the mouse, stained using phalloidin to label actin (green) and DRAQ5 to label DNA (blue). The oocyte in the centre is delimited by the actin-rich cortex. Two layers of granulosa cells surround it, and the dense forest of hair-like actin-rich TZPs is easily seen. Note that TZPs appear to project from all granulosa cells in the innermost layer. Reproduced with permission from ( Clarke, 2017b ). B) The number of actin-rich TZPs (left) increases substantially as oocytes grow. Tubulin-rich TZPs are much less numerous and do not detectably increase in number during oocyte growth. Reproduced with permission from ( El-Hayek *et al*., 2018 ). C) Gap junctions become assembled where the tips of the TZPs contact the oocyte plasma membrane, and these permit the transfer of metabolites that support oocyte growth as well as of cyclic GMP that prevents initiation of meiotic maturation. Transfer from the growing oocyte to the granulosa cells is possible in principle but has not been described. Adapted with permission from ( Clarke, 2017b ). D) Adherens junctions maintain contact between the TZPs and the oocyte. Note that different cadherins appear to be principally responsible in the two cell types. Embryonic poly(A)-binding protein (EPAB) and the focal adhesion kinase, proline-rich tyrosine kinase 2 (PTK2), may regulate translation of the mRNA encoding E-cadherin and assembly of the adherens junction, respectively.

Our understanding of TZPs took another leap forward with the advent of confocal microscopy, which provided both improved resolution and, compared to traditional fluorescence microscopy, the ability to image optical sections within the large granulosa cell-oocyte complex. Studies by David Albertini and colleagues, as well as other groups, established that many TZPs contained a backbone of actin ( Fig. 3A ; Albertini and Rider, 1994 ; Albertini *et al*., 2001 ; Barrett *et al*., 2010 ; Li and Albertini, 2013 ; Macaulay *et al*., 2014 ; McGinnis and Kinsey, 2015 ; El-Hayek *et al*., 2018 ). This anatomical feature together with their size suggests that TZPs are a type of filopodium. In support of this, we recently showed that the two types of membrane protrusions share structural elements, including DAAM1, a member of the formin family of proteins that nucleate actin assembly and fascin, which bundles actin filaments to produce stiffer fibres ( El-Hayek *et al*., 2018 ). As oocytes grow and become enclosed by an increasing number of granulosa cells, corresponding to the increase in its surface area, the number of actin-rich TZPs steadily increases ( El-Hayek *et al*., 2018 ).

In addition to the actin-rich TZPs, there exists also a population of TZPs possessing a core or backbone of tubulin. In mice, the tubulin-rich TZP population is very small – about two orders of magnitude less abundant than the actin-rich population ( Fig. 3B ; El-Hayek *et al*., 2018 ). In other mammals including primates, however, studies indicate that the tubulin-rich population may be much larger ( Allworth and Albertini, 1993 ; Albertini and Rider, 1994 ). It would be valuable to quantify the tubulin-rich TZP population in non-rodent species to determine whether this reported difference reflects biological or methodological differences.

## Function of TZPs

As discussed above, it was recognized as soon as TZPs were identified that they might play a key role in supporting the oocyte during its development ( Anderson and Beams, 1960 ; Zamboni, 1974 ; Albertini *et al*., 2001 ). More recent studies have enabled this role to be experimentally established and molecularly defined. TZPs likely play both a nutritional and a structural role during oocyte development ( Fig. 3C, D ). The function of the gap junctions that become assembled where the tips of the TZPs contact the oocyte plasma membrane has been extensively investigated and is well-understood ( Phillips and Dekel, 1991 ; Sela-Abramovich *et al*., 2006 ; Kidder and Vanderhyden, 2010 ; Wigglesworth *et al*., 2013 ; Winterhager and Kidder, 2015 ). Studies using radioactive tracers showed that sugars, amino acids and nucleotides were transferred from the granulosa cells to the oocyte ( Eppig, 1979 ; Brower and Schultz, 1982 ). Notably, the mouse oocyte contains small quantities of mRNAs encoding enzymes that convert glucose to pyruvate and of the RNA encoding the transporter that mediates uptake of extracellular alanine ( Eppig *et al*., 2005 ; Sugiura *et al*., 2005 ). It should be noted that pyruvate can be transferred either through gap junctions or extracellularly. When *Gja4*, encoding the principal component of gap junctions in oocytes, also termed connexin 37, was knocked out in the mouse, the oocytes failed to grow to full size and were not able to undergo meiotic maturation ( Simon *et al*., 1997 ; Carabatsos *et al*., 2000 ; Gittens *et al*., 2005 ). Although not formally ruling out a gap junction-independent role for GJA4, this observation highlights the essential nature of the physical communication between the growing oocyte and surrounding somatic cells.

When oocytes have reached full size, they are able to undergo meiotic maturation, but are prevented from doing so by cyclic GMP that is synthesized by the granulosa cells and transferred to the oocyte via gap junctions ( Norris *et al*., 2009 ; Zhang *et al*., 2010 ; Jaffe and Egbert, 2016 ). Conversely, following the ovulatory release of luteinizing hormone, cGMP level rapidly falls in the granulosa cells – as a result the nucleotide flows back to the granulosa cells from the oocyte along a concentration gradient, which enables maturation to begin in the oocyte. Thus, the TZPs permit essential communication that regulates both oocyte growth and meiotic maturation.

TZPs also provide the substrate for adhesion between the oocyte and granulosa cells ( Fig. 3D ). Immunofluorescence studies have shown that granulosa cells express N-cadherin including on the TZPs, whereas oocytes express mainly E-cadherin ( Mora *et al*., 2012 ; Lowther *et al*., 2017 ). As well, focal adhesion kinase (FAK, also known as PTK) which participates in the formation and maturation of adherens junctions is also abundantly expressed in the granulosa cell immediately adjacent to the *zona pellucida* as well as the oocyte ( McGinnis and Kinsey, 2015 ; see also below). These observations, together with the membrane localization of β-catenin, suggests that adherens junctions mediate the cell adhesion. In contrast, classical markers of desmosomes have not been detected ( Mora *et al*., 2012 ), implying that this type of intercellular junction is not present. It has been speculated that the adherens junctions are present in the tubulin-rich TZPs, where the gap junctions are found in the actin-rich TZPs ( Li and Albertini, 2013 ), indicating that the two types of TZPs perform different duties.

Oocytes are able to grow partially when detectable gap junctional communication is blocked but not at all when they are physically separated from the granulosa cells ( Eppig, 1979 ; Simon *et al*., 1997 ; Gittens *et al*., 2005 ; Li *et al*., 2007 ; Clarke, 2017b ). This implies that granulosa cell-oocyte adhesion serves additional roles during oocyte growth, independently of permitting gap junctional coupling. Kit ligand (KL) produced by the granulosa cells can promote oocyte growth *in vitro* and it may do the same *in vivo* ( Packer *et al*., 1994 ; Elvin *et al*., 1999 ; Thomas *et al*., 2008 ). The granulosa cells produce mRNAs encoding both soluble and trans-membrane forms of KL ( Joyce *et al*., 1999 ; Thomas *et al*., 2005 ; Demeestere *et al*., 2012 ). If the trans-membrane form is the main bioactive form *in vivo*, as suggested by the *in vitro* data, then it must be located at the position where the TZPs contact the oocyte plasma membrane. This in turn implies that a mechanism may exist to transport newly synthesized KL to the tips of the TZPs. Conversely, oocyte-derived factors, including growth-differentiation factor 9 (GDF9) and bone morphogenetic protein 15 (BMP15), regulate the differentiation of the granulosa cells ( Peng *et al*., 2013 ; Mottershead *et al*., 2015 ; Clarke, 2017b ). Although GDF9 and BMP15 can exert known physiological functions (for example, expansion of the cumulus cell layer) when supplied in soluble form, and membrane-bound variants have not been described for either, it is intriguing to speculate whether, *in vivo* , they may be presented to the granulosa cells in a membrane-associated context. Such a model implies that their receptors would be enriched at the tips of the TZPs, a prediction that could be experimentally tested.

Recent studies have identified small vesicles lying between the tips of TZPs and the plasma membrane of the oocyte. In addition, transport of mRNA from the granulosa cell bodies to the tip of TZPs, as well as its transfer to the oocyte, was also observed ( Macaulay *et al*., 2014 , 2016 ). Based on these results, the authors suggest that, by budding off vesicles that subsequently fuse with the oocyte plasma membrane, the granulosa cells can supply the oocyte with macromolecules and organelles that are much too large to be transferred through gap junctions.

## Mechanism of TZP formation

**Figure 4:**
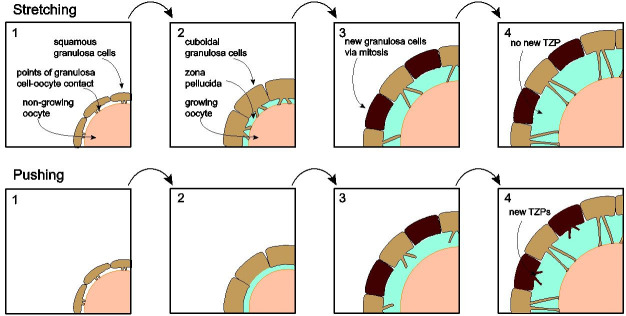
Models of TZP formation. Upper: stretching model. Oocytes and granulosa cells in primordial follicles establish cell junctions. When the oocyte begins to grow, it elaborates the *zona pellucida*. As the thickening matrix pushes the granulosa cell bodies from the oocyte, they remain attached at the original junctional connections, thus producing a stretched cytoplasmic filament. As oocytes grow, the new granulosa cells (dark brown) generated by mitosis so that the somatic cells continue to fully cover the expanding oocyte surface will not have TZPs linking them to the oocyte. Lower: pushing model. After deposition of the *zona pellucida*, the granulosa cells elaborate TZPs that push through the matrix to reach the oocyte surface. All granulosa cells of the innermost layer generate TZPs. The two models are not mutually exclusive.

How are TZPs generated? Two mechanisms may be considered, which may be termed stretching and pushing ( Fig. 4 ). The stretching mechanism derives from two major observations. First, in primordial follicles, there is no *zona pellucida* separating the oocyte and granulosa cells. After the oocytes begin to grow within primary follicles, it is deposited in discrete ‘chunks’ that eventually become joined together to establish an intact structure ( Chiquoine, 1960 ; Zamboni, 1974 ). Second, prior to formation of the *zona pellucida*, intercellular junctions link the oocyte to its neighboring granulosa cells ( Mora *et al*., 2012 ). Thus, it can be envisioned that the *zona pellucida* becomes established around pre-existing points of contact between the oocyte and granulosa cell and, as the *zona pellucida* thickens, pushing the granulosa cells away from the oocyte, the cytoplasm stretches to maintain the point of contact ( Chiquoine, 1960 ; Hadek, 1965 ; Hope, 1965 ).

Although this model is attractively simple, it is not easily reconciled with several characteristics of TZPs. First, as reported by several groups based on observation and recently quantified by us, the number of TZPs increases by at least an order of magnitude during oocyte growth ( Makabe *et al*., 2006 ; Li and Albertini, 2013 ; El-Hayek *et al*., 2018 ). Second, because the *zona pellucida* is laid down early during oocyte growth, many granulosa cells adjacent to the oocyte must be born after this event. According to the stretching model, these would not possess TZPs; yet TZPs appear to project from every granulosa cell that lies adjacent to the *zona pellucida*. Third, TZPs are destroyed when follicles are cryopreserved, yet regenerate when they are thawed and placed in culture ( Barrett *et al*., 2010 ). These observations cannot easily be accommodated by the stretching model.

The pushing model holds that TZPs grow out from granulosa cells, in a manner possibly analogous to filopodial growth, and penetrate through the *zona pellucida* to reach the oocyte. This model could account both for the continuous increase in the number of TZPs and for their presence on all *zona pellucida*-adjacent granulosa cells. To test it, we combined wild-type oocytes that were fully enclosed by a *zona pellucida* but were completely stripped of granulosa cells with a mixture of wild-type and fluorescently tagged granulosa cells and incubated the reaggregates ( El-Hayek *et al*., 2018 ). After several days, we observed fluorescently tagged TZPs projecting from tagged granulosa cells to the oocyte. These could only have been generated after we constructed the reaggregates. To test whether these projections were functional, we injected a gap junction-permeable dye into the oocyte and observed that it spread to the neighbouring granulosa cells. These results conclusively demonstrated that granulosa cells are able to elaborate TZPs de novo that can penetrate through the *zona pellucida* to reach the oocyte.

Although the both theoretical and experimental evidence suggest that most TZPs are formed de novo, the two models are not entirely incompatible. It is plausible that some TZPs are derived from the points of granulosa cell-oocyte adhesion that existed prior to elaboration of the *zona pellucida*. In this context, it may be recalled that there exists a small population of tubulin-rich TZPs. It could be speculated that this sub-population of TZPs is generated through the stretching mechanism.

What signals trigger TZP formation by the granulosa cells? As described above, GDF9 and BMP15 produced by the oocyte regulate differentiation and gene activity in granulosa cells. Strikingly, in *Gdf9* ^-/-^ mice, fewer TZPs are found and their structure is disorganized ( Carabatsos *et al*., 1998 ). Moreover, when GDF9 was depleted in wild-type oocytes, through injection of RNAi, the granulosa cells subsequently generated fewer TZPs ( El-Hayek *et al*., 2018 ). These results link GDF9 produced by the oocyte to formation of TZPs by the granulosa cells. GDF9 also up-regulates the expression of genes encoding DAAM1 and fascin, as well as MYO10 which promotes filopodial growth, suggesting a possible mechanism for its TZP-promoting activity ( El-Hayek *et al*., 2018 ). Conversely, it has been reported that the granulosa cells of mice lacking follicle-stimulating hormone (FSH) possess more TZPs than those of wild-type mice, and that administration of FSH decreases TZP number ( Combelles *et al*., 2004 ). This may indicate that, once oocytes have reached full-size and become competent to develop as embryos, TZP-number decreases by an FSH-dependent mechanism ( Li and Albertini, 2013 ).

Based on the directionality of TZP growth towards the oocyte, it is tempting to speculate that the oocyte provides chemotactic cues that guide the TZP. It is also possible, however, that there is no preferred direction for TZP growth, which instead occurs wherever it can do so unimpeded. In this context, it is noteworthy that, at the time of formation of primordial follicles, the pre-granulosa cells extend long arms that enclose the oocyte ( Pepling and Spradling, 2001 ; Lechowska *et al*., 2011 ). While these arms are much larger than TZPs, this observation raises the possibility that (pre-) granulosa cells are predisposed to generate cytoplasmic projections including TZPs.

Once the newly elaborated TZP reaches the oocyte surface, the two plasma membranes become associated by adherens junctions, as discussed above, and gap junctions are assembled to metabolically couple the cells. The mechanisms by which these junctions are assembled, as well as whether the TZP-oocyte membrane interaction is stable or dynamic, remains unknown. When *Ptk2* was deleted from growing oocytes, the number of TZPs as well as gap junctional communication were strikingly reduced. This indicates that PTK2 in the oocyte is required to establish or maintain TZP-oocyte contact ( McGinnis and Kinsey, 2015 ). Additionally, deletion of *Epab*, encoding an RNA-binding protein that promotes translation, in oocytes caused a decrease in the quantity of E-cadherin in the oocyte and in the number of TZPs ( Lowther *et al*., 2017 ). These results suggest that E-cadherin may be required to anchor TZPs to the oocyte plasma membrane.

## Questions for the future

Although the basic structure and function of TZPs have long been known, the mechanisms by which these indispensable vehicles of communication between the developing oocyte and its follicular environment are produced have remained mysterious. Recent results establish that they are continuously produced by the granulosa cells surrounding growing oocytes. In view of observations that granulosa cell-oocyte communication is impaired in certain disease conditions associated with infertility ( Ratchford *et al*., 2008 ) and that TZP numbers are reduced in aging females ( El-Hayek *et al*., 2018 ), a better understanding of how TZPs are established and maintained is crucial for improving assisted reproduction in agricultural and clinical contexts. Questions to be answered include:

1. Are TZPs dynamic or stable structures? If dynamic, what controls the relative rates of elaboration and retraction? Does the stability of a TZP depend on the cell-cycle state of the granulosa cell?
2. Are TZPs actively guided towards the oocyte or do they simply grow where there is space?
3. Is the *zona pellucida* sufficiently porous to permit growing TZPs to penetrate through it or must they digest a path?
4. Do granulosa cells stop generating TZPs once oocytes have reached full size? If so, by what mechanism?
5. Many studies have reported that TZPs are lost during meiotic maturation ( Gilula *et al*., 1978 ; De Smedt and Szollosi, 1991 ; Phillips and Dekel, 1991 ; Allworth and Albertini, 1993 ; Suzuki *et al*., 2000 ; Barrett and Albertini, 2010 ). What is the mechanism?

The answers to these and other questions will not only contribute to understanding how the growing oocyte interacts with its environment but will also uncover principles governing the regulation of intercellular contact and communication.
